# A Model-Based Meta-Analysis of Willingness to Participate in Cancer Screening

**DOI:** 10.3390/ijerph18052580

**Published:** 2021-03-04

**Authors:** Guangchao Charles Feng, Zhiliang Lin, Wanhua Ou, Xianglin Su, Qing Yan

**Affiliations:** 1 College of Communication, Shenzhen University, Shenzhen 518600, China; winnie.ouwh@hotmail.com (W.O.); 2170094510@email.szu.edu.cn (X.S.); 2 School of Literature and Media, Nanfang College of Sun Yat-sen University, Guangzhou 510970, China; linzhl@nfu.edu.cn; 3 School of Journalism and Communication, Jinan University, Guangzhou 510610, China; tyq2008@jnu.edu.cn

**Keywords:** cancer screening, model-based meta-analysis, health belief model

## Abstract

Although early screening tests are beneficial for the detection and treatment of cancers, many people have failed to participate in screening tests. The present study aims to explore the theoretical underpinning of low participation in screening programs using the method of meta-analytic structural equation modeling. It was found that the health belief model is the most adopted theoretical framework. Moreover, the intended uptake of screening was positively predicted only by cues to action, health literacy, and perceived susceptibility. As a result, a health intention model, including the three significant variables, is proposed. The practical implications of the findings are that health communication campaigns should focus on enlightening and engaging the public through all necessary means to raise awareness and transfer knowledge in relation to screening procedures as well as cancers per se.

## 1. Introduction

Cancer is one of the leading causes of morbidity and mortality worldwide [1] (the primary studies included in the present meta-analysis are marked with asterisks in the References section). However, a substantial proportion of cancer-related deaths could be prevented [2] if the early detection of cancer, such as through medically warranted cancer screening, could be undertaken [3]. Cancer screening in the present study is defined as detecting cancer at an asymptomatic stage of development using medical screening services [3,4]. Consequently, some means of prevention, such as self-examination of breast and using self-purchased medical kits, are irrelevant. A high number of screening trials have shown the efficacy of cancer screening in reducing cancer-specific mortality for cancers of the breast, colon, mouth, skin, rectum, larynx, cervix, and lung [2,3,5,6]. Consequently, measures of early cancer detection, such as mammography associated with breast cancer, colonoscopy, sigmoidoscopy, high-sensitivity fecal occult blood tests (FOBTs) associated with colorectal cancer, low-dose helical computed tomography associated with lung cancer, and Pap test and human papillomavirus (HPV) testing associated with cervical cancer, are strongly recommended (for the recommendations of the United States Preventative Services Task Force (USPSTF) regarding cancer-screening practices, see USPSTF [7,8,9,10,11]).

Despite the fact that early screening tests are beneficial for the detection and treatment of cancers, many people in the recommended age groups [7,12,13] have failed to participate in screening tests [4,14]. Low participation in screening programs has been attributed to patient, health professional, and organizational factors [3], specifically including the lack of knowledge [15,16] and physician recommendations [17], health insurance status [18], socioeconomic inequalities [17,19], and a variety of barriers [20,21]. These are all important factors, but a premium has to be placed on the elucidation of theoretical models of preventive health behavior [22]. As some [23,24] have pointed out, it is urgent to develop and adopt theory-based intervention programs to increase healthy behaviors. Valid theories can not only consistently explain why a phenomenon persists to develop effective interventions but also predict what will happen in the future to take preventive measures (see [25]). Studies that address cancer screening behavioral intentions are abundant but often lack enough integration of the relevant theories. According to our discursive search in major databases, at least 14 theories have been applied in this area (for a review, see [3]). Among them, the health belief model (HBM) [26,27], whose extensions account for 40% of the studies in our sample, is the most popular, followed by the theory of reasoned action (TRA) [28,29] and its extension, namely, the theory of planned behavior (TPB) [30]. Due to its popularity, the HBM will be primarily employed in the following analyses, whereas its connections with other closely related models are duly addressed.

The research purpose of the present study is twofold. One is to estimate the common effect sizes explaining cancer-screening intention and behaviors, and the other one is to find and examine a conceptual model that can adequately account for cancer-screening intention and behaviors. The first purpose is usually achieved by conventional univariate meta-analysis, whereas we will conduct an innovative model-based meta-analysis using the technique called meta-analytic structural equation modeling (MASEM) to achieve the two objectives sequentially.

## 2. Literature Review

### 2.1. Health Belief Model (HBM) and Its Extensions

The HBM [31] was originally formulated in response to the failure of a free tuberculosis (TB) health-screening program and was later developed to account for a variety of both long- and short-term preventive health behaviors. The HBM [26,27,32,33] hypothesizes that a person will take a health-related action if the individual perceives a severe negative health outcome (perceived severity), feels susceptible to it (perceived susceptibility), perceives high benefits of undertaking the preventive behavior (perceived benefits), or perceives low barriers to adopting those behaviors. In addition, the model also includes a cue to action whereby the individual is driven to engage in preventive behavior. Cues to action could include external cues, such as a public service announcement or interpersonal interaction, or internal cues such as the perception of an unsound bodily state [26,32,33].

Some researchers [34,35,36] modified the HBM while hypothesizing that screening intentions are directly influenced by the factor of perceived benefits, which are directly influenced by the susceptibility to the health threat and the perceived severity of the threat, and the costs of the action. The HBM later included the component of self-efficacy [37] as well as the incentive to behave (health motivation) [38].

### 2.2. Theory of Reasoned Action (TRA) and Theory of Planned Behavior (TPB)

The health belief model (HBM) essentially explains the relationships between both internal and external health beliefs and health behaviors (or intentions), whereas such generalized connections are well documented in the TRA and its extension, namely, the TPB. The TRA has also been adopted to study various types of cancer screening behavioral intentions [39,40,41], and some expanded TRA has been employed for the same purpose. For instance, Montano and associates [39,42] incorporated affect, habit, and facilitating conditions into their expanded TRA to predict mammography participation.

Many also have applied the TPB to examine participation in cancer screening [43,44,45,46]. Despite its popularity, Gaston and Gerjo [47] reported that the TPB could explain 41% of the variance in intention but only 34% of the variance in a variety of health behaviors. Therefore, some have made extensions to the TPB by including more factors to better account for actual behavior. For instance, Conner and Armitage [48] extended the TPB by including past behavior and habit, belief salience, affect, self-identity, and moral norms, among others (also see [49]). The information–motivation–behavioral skill model (IMB) [50] expanded the TPB by adding the component of functional cancer literacy, which includes three domains: breast cancer awareness, knowledge and screening, and prevention and control.

### 2.3. Research Questions

Dissimilar theories with numerous predictors or similar theories with diverse combinations and configurations of predictors have been adopted to the same end, i.e., to account for the determinants of preventive health behavior. Some predictors were found to be important in some studies, but contrary conclusions were found in other studies. Consequently, a meta-analysis, which is a means of quantitatively estimating the overall effect based on previous research findings on a particular topic [51,52,53], is intended to clarify this confusion. Due to its popularity, several univariate meta-analyses have been used with the health belief model (HBM) [54,55,56,57] or the HBM equivalent [58]. The meta-analysis of Carpenter [55] also found that barriers and benefits were strong predictors, but severity and susceptibility were not. In their meta-analysis, Harrison, Mullen and Green [54] concluded that the factor of costs (barriers) is a relatively more critical predictor, whereas the effect of severity is negligible, and benefits and susceptibility play a minor role in contributing to screening behavior. In the meta-analysis of the protection motivation theory (PMT), which is a variant of the HBM, Milne, Sheeran and Orbell [58] found that the association between threat appraisal (severity, vulnerability, and fear) and intention was small, whereas the associations between the coping-appraisal variables (self-efficacy, and costs or barriers) and intention were moderate. Moreover, all of these predictors had weaker associations with behaviors than with intentions. Therefore, the inconsistency in the findings remains among these meta-analyses.

The correlations and complex relationships (e.g., moderation and mediation) in any theoretical models will affect the magnitude and the standard error of the statistic for testing the relationship between the predictor of interest and the dependent variable. Therefore, univariate meta-analysis as reviewed above inevitably shares the deficiency of primary studies, i.e., failing to shed light on the true relationships among the variables of a theory. In fact, few primary studies merely examined the bivariate correlation in actual studies but rather addressed more complex theoretical relationships through incorporating either covariates, moderators, or mediators [59]. Model-based as opposed to separate univariate correlation-based meta-analysis is a technique that can be used to analyze complex chains of events [60] and hence is adopted in the present study. Furthermore, due to the correspondence among the aforementioned value-expectancy-based theories, a meta-analysis focusing on any one of them would just prioritize idiosyncrasies but would be subject to the loss of generality. Accordingly, the present study elects to seek all of the pieces of the theoretical framework by studying the common outcome using the model-based meta-analysis, i.e., the behavior and intentions of participating in cancer screening, and subsequently form a variety of combinations of the pieces; that is, the main purpose of the meta-analysis study is not to confirm the hypotheses stipulated in the relevant theories or models, so we will raise the following research questions rather than hypotheses:

1. What are the magnitudes of effects of perceived severity, perceived barriers, perceived benefits, health literacy, cues to action, perceived susceptibility, and perceived behavioral control on cancer-screening intentions and behaviors?
2. What kinds of significant relationships among the predictors mentioned in the first research question and outcomes (cancer-screening intentions and behaviors) are present? Which theories underpin these relationships?

## 3. Methods

### 3.1. Classification of Constructs

Based on the “core health cognitions” framework of McMillan and Conner [61], we identified the major constructs associated with the abovementioned theories and then consolidated these constructs into nine core categories: cues to action, health literacy, norms, perceived behavioral control, risk perceptions (including perceived severity and susceptibility), perceived barriers, intentions, and screening behavior. We inspected the variables’ underlying meanings in the included studies and allocated the variables to the nine construct categories on a “close fit” basis [24].

### 3.2. Selection Criteria

**Figure 1:**
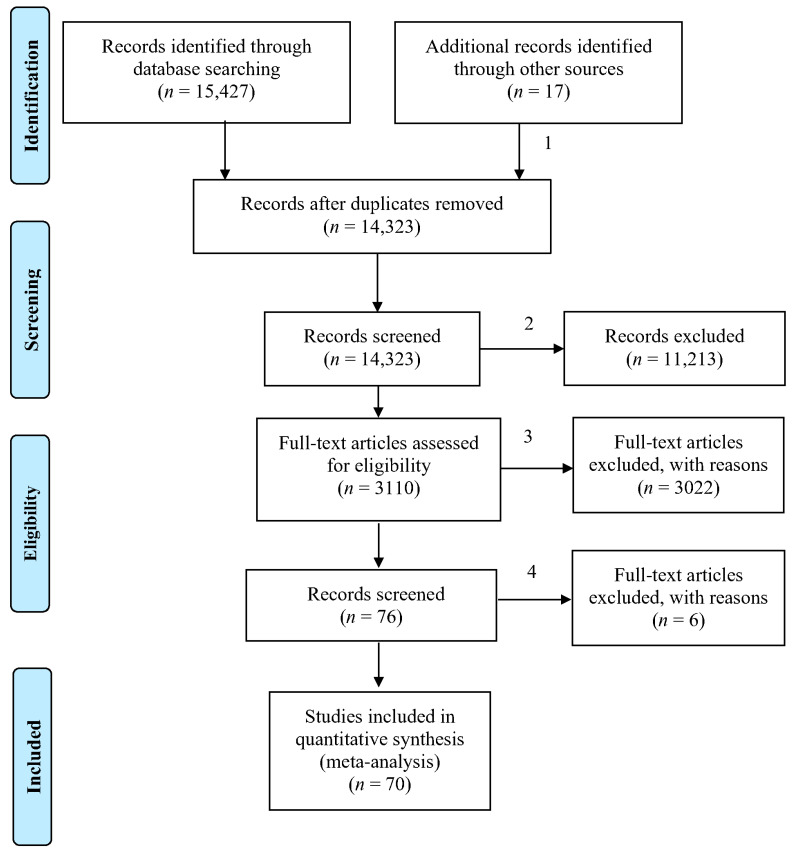
PRISMA 2009 Flow Diagram (PRISMA 2009 Flow Diagram. Note: (1) Contacted the studies’ corresponding authors without providing needed information and searched through the reference lists of the located studies; (2) articles that were not quantitative studies and those without theoretical variables concerning the determinants and compliance or intentions of cancer screening were filtered out; (3) articles without reporting complete zero-order correlations among the independent variables and dependent variables were filtered out; and (4) the articles on prostate cancer and one study with a large sample size were removed because the United States Preventative Services Task Force (USPSTF) recommendations for prostate cancer screening have changed).

Since cancer-screening intentions and behaviors are the focus of our study, we tried combinations of the following keywords, namely, “screening tests for *cancer*”, “cancer* screening”, “screening for * cancer*”, “intent* cancer”, “participate* cancer”, “compl* cancer”, and “adher* cancer”, in eight different databases, including EMBASE, Cochrane Database of Systematic Reviews, PsycINFO, PubMed, Web of Science, Scopus, and Google Scholar, for published articles related to various factors associated with participation in cancer screening. The searching and selecting process followed the PRISMA (Preferred Reporting Items for Systematic Reviews and Meta-Analyses) recommendations (see Figure 1), and the three research assistants of the first author finished the process. The first round of the search started in November 2019, yielding 12,427 potentially eligible studies. We then made a screening of these articles. The selection criteria for the studies to be included in this meta-analysis were as follows: (a) quantitative studies with effect sizes; (b) articles with theoretical variables concerning the determinants and adherence or intentions of cancer screening (studies using only medical variables were hence excluded); and (c) reporting complete zero-order correlations among independent variables and dependent variables. After a series of filtered searches, we obtained 56 eligible articles.

For those articles not satisfying criterion c, we contacted the corresponding authors to request the missing information. This step retrieved an additional 10 eligible studies. We additionally searched through the reference lists of all located studies and obtained eight eligible articles. The final count of the total number of relevant eligible articles reached 70 with 3288 valid effect sizes (cumulative *n* = 754,294).

### 3.3. Unit of Analysis

The unit of analysis is the effect size, which is the correlations (Pearson’s or other types of correlations that are appropriate for other measurement levels) between the intention to participate in cancer screening and a few predictors. The experts of MASEM [59,62] do not recommend the method of Fisher’s z transformation and back-transformation in MASEM. Consequently, in the following analyses, the original correlation will not be transformed into Fisher’s z, a popular procedure termed the Rosenthal [53] approach by Johnson et al. [63], in order to retain the correlation metric and the associated variances and covariances among the correlations for use in Stage 2 of MASEM.

### 3.4. Coding Categories of the Moderators

Differences in the methods and sample characteristics may introduce variability (“heterogeneity”) among the true effects. Therefore, once heterogeneity is detected, the moderator analysis is imperative. The following information was coded from each article: (a) date of publication (range = (1990, 2017), 84.507% of studies were conducted after 2000); (b) number of observations (M = 1256, SD = 4624); (c) journal names; (d) data types (cross-sectional (85.915%) vs. time series); (e) sampling types (random or probability sampling (66.197%) vs. convenience sampling); (f) country of study (countries were classified into individualistic (81.690%) vs. collectivistic categories according to Hofstede [64]); and (g) cancer types (breast cancer, colorectal cancer, cervical cancer, both breast and cervical cancer, and a general type without specifying the name accounted for 24 (33.803%), 19 (26.760%), 8 (11.268%), 19 (26.760%), and 1 (1.408%), respectively).

### 3.5. Procedures

**Figure 2:**
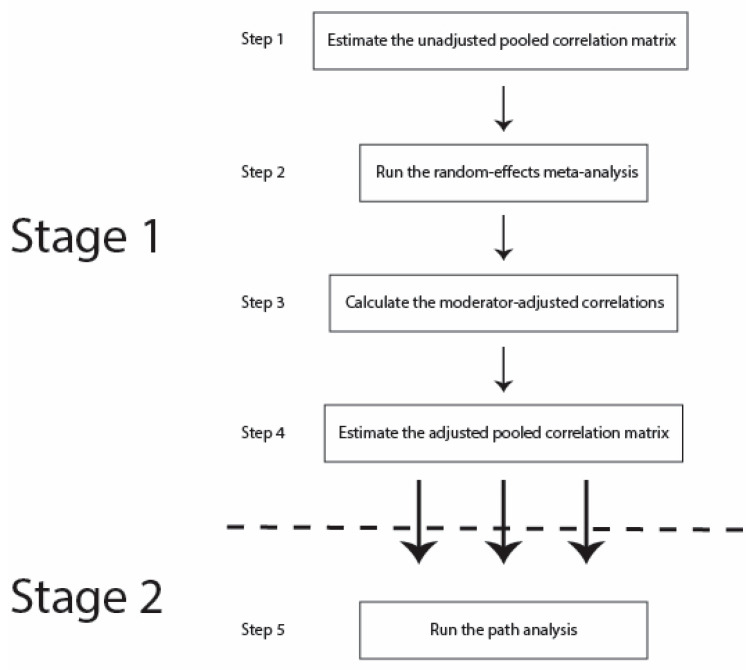
Procedures of MASEM.

Overall, there were five steps in the present study (see Figure 2). Step 1 through Step 4 form Stage 1 of the so-called two-stage MASEM approach, leaving Step 5 in Stage 2. However, unlike Cheung and Chan [65], who assumed that all studies have the same population correlation matrices based on fixed-effects models [66], the present study estimated the heterogeneity using a homogeneity statistic *Q* [67,68,69,70] in Stage 1 [71]. In the absence of homogeneity, the random-effects model (as opposed to a fixed-effects model, which assumes that the true effect is the same for all studies), which allows the true effect to vary across studies, was used [68,69,72]. Furthermore, we accounted for variability in heterogeneous effect sizes by relating them to the studies’ coded attributes; that is, the pooled correlation matrix is adjusted by the moderator effect. The fitted models were estimated based on the Akaike information criterion (AIC), followed by the QE (test statistic of residual heterogeneity) and QM (omnibus test statistic of the significance of moderators) statistics.

In addition, the compilation of effect sizes showed a clear hierarchical structure, as there were multiple effect sizes for many studies. Consequently, to sidestep the dependence problem among the effect sizes, we analyzed these data with multilevel mixed-effects modeling using the metafor package (version 2.10) of the R language (version 3.51) [73], which is generally superior to other approaches, e.g., robust variance estimation and averaging effect sizes [59,74,75]. It should be noted that this practice in Stage 1 is also different from the procedure proposed by Cheung [65,76].

The two-stage MASEM approach that the present study performs consists of the following five steps:

Step 1: The first step involves the estimation of a pooled correlation matrix of the relations. A matrix is constructed to represent the zero-order relations among all of the nine variables (see Table 1). The unadjusted pooled correlation matrix and the heterogeneity are estimated using the random-effects meta-analysis with the multilevel modeling approach. The cell number indicating the position of a particular correlation in the original correlation matrix is tested as a moderator in the model without the intercept. The resulting coefficients are the pooled correlation matrix.

Step 2: The next step is again to run the random-effects meta-analysis in the multilevel model with the intercept, taking into account the aforementioned study moderators.

Step 3: The resulting meta-regression coefficients and associated residual values in the above meta-regression model are used to calculate the moderator-adjusted correlations.

Step 4: Estimate the adjusted pooled correlation matrix in the same way as in Step 1 but replace the original correlation coefficients with the adjusted ones derived in Step 3.

Step 5: The path models involving the predictors of the intention and behavior based on the HBM and relevant theories based on the adjusted pooled correlation matrix are estimated using the weighted least squares (WLS) estimation (with 5000 parametric bootstrap replicates) of metaSEM [77].

## 4. Results

**Figure 3:**
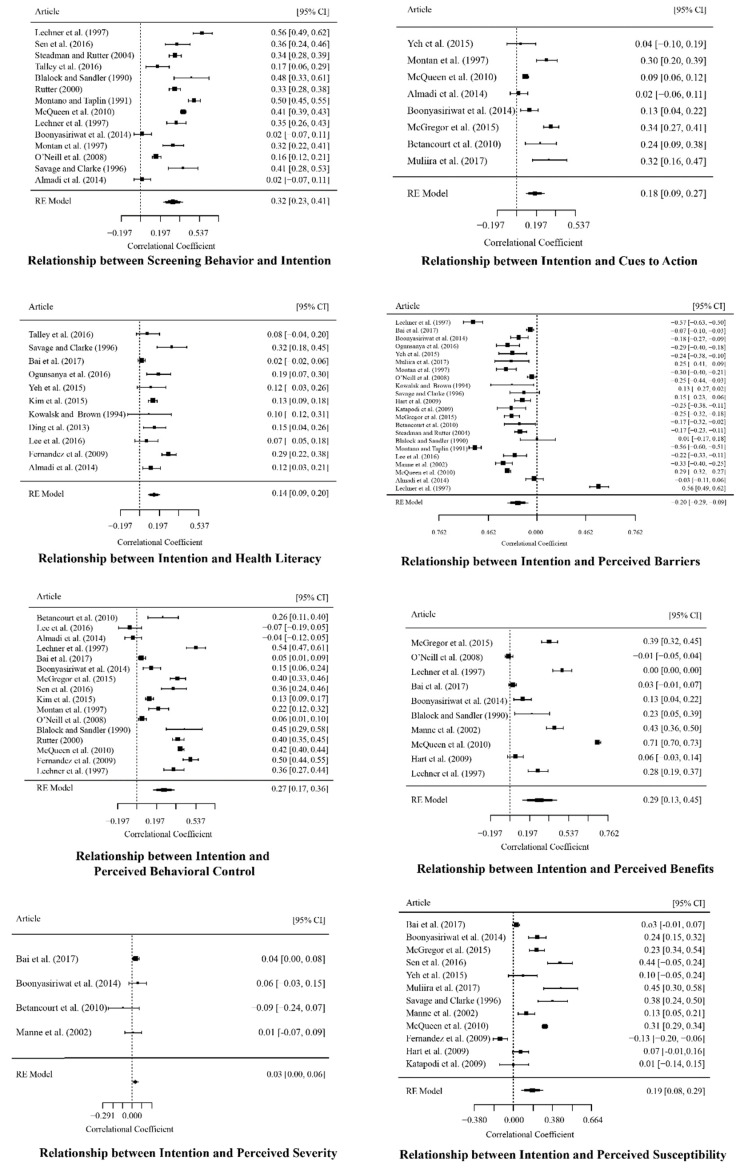
Forest plots [34,39,42,45,46,81,82,83,84,85,86,87,88,89,90,91,92,93,94,95,96,97,98,99,100,101,102,103,104,105,106,107,108,109,110,111,112,113,114,115,116,117,118,119,120,121,122,123,124,125,126,127,128,129,130,131,132,133,134,135,136,137,138,139,140,141,142,143,144,145].

**Figure 4:**
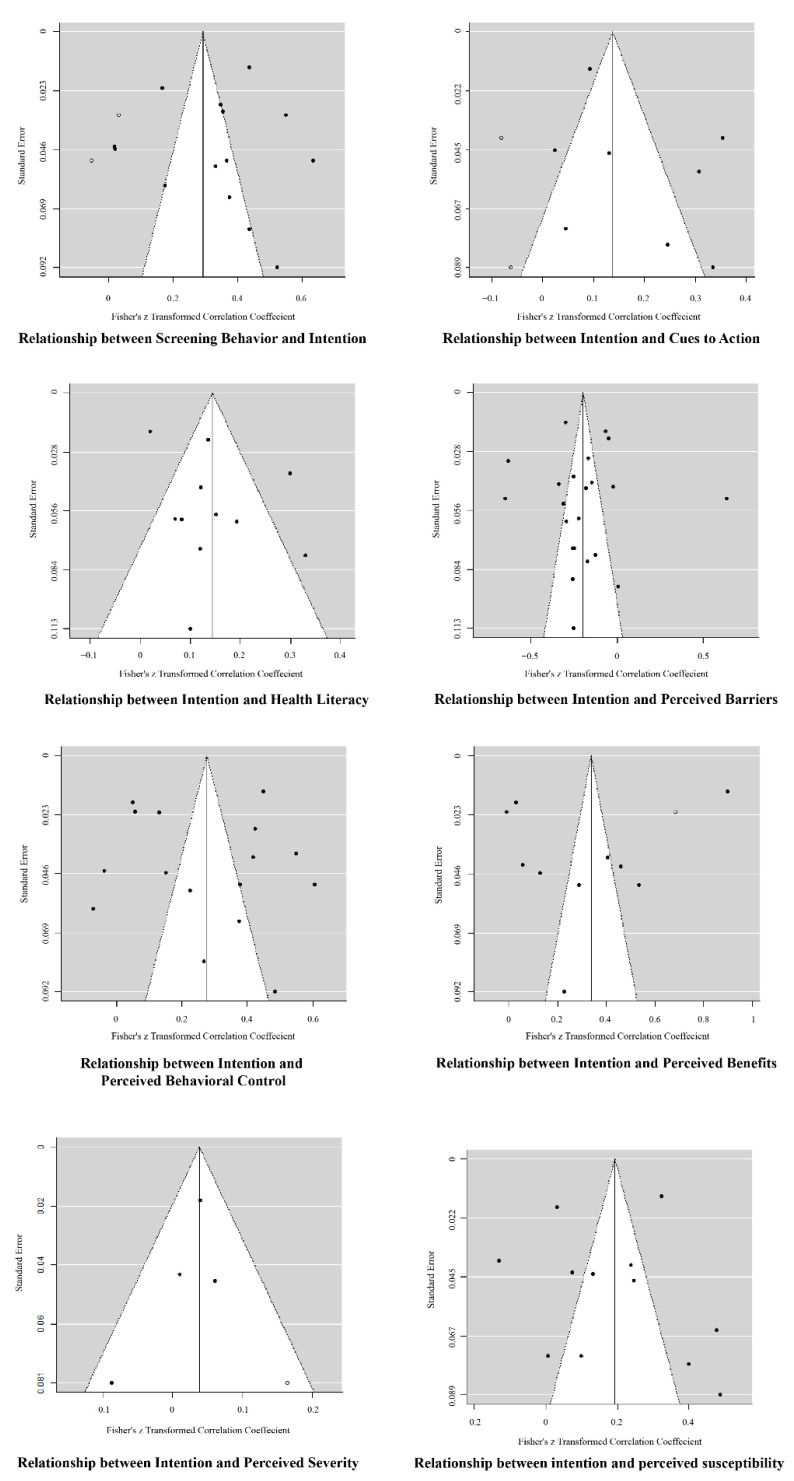
Funnel plots (solid circles represent the weight of the studies, and empty circles represent the added studies).

The multilevel modeling in Step 1 was performed to derive the pooled correlation matrix (shown in Table 1). The test for residual heterogeneity (QE (df = 1067) = 21,928.921, *p* < 0.001) and the test of moderators (QM (df = 36) = 675.245, *p* < 0.001) were both significant. The intraclass correlation (ICC) value is 0.157, which indicates that 15.70% of the variance is attributable to the between-study variability. Based on the pooled correlation matrix derived from Step 1, the univariate effect sizes of interest, whose results correspond to the first research question, can be extracted. As shown in Table 1, there generally exist weak effect sizes for the predictors on screening intentions ($r_{b}$ = −0.116, *p* < 0.001; $r_{INT\_PBAR}$ = 0.267, *p* < 0.001; $r_{INT\_PBC}$ = 0.212, *p* < 0.001; $r_{PSEV\_INT}$ = 0.029, n.s.; $r_{PSUS\_INT}$ = 0.181, *p* < 0.001; $r_{CTA\_INT}$ = 0.166, *p* < 0.001; $r_{LIT\_INT}$ = 0.158, *p* < 0.001). In addition, the effect sizes of the predictors on actual screening behavior showed similar results to those of the predicting intentions ($r_{CS\_PBAR}$ = −0.147, *p* < 0.001; $r_{CS\_PBEN}$ = 0.196, *p* < 0.001; $r_{CS\_PBC}$ = 0.164, *p* < 0.001; $r_{CS\_PSEV}$ = 0.031, n.s.; $r_{CS\_PSUS}$ = 0.071, *p* < 0.01; $r_{CS\_CTA}$ = 0.167, *p* < 0.001; $r_{LIT\_CS}$ = 0.145, *p* < 0.001; $r_{CS\_INT}$ = 0.259, *p* < 0.001). Subsequently, the forest plots (see Figure 3) indicating the estimated common effects as well as the intervals, visually display the results of Table 2. Furthermore, the funnel plots (see Figure 4), demonstrating possible publication bias, are also presented. According to the funnel plots based on the trim-and-fill analysis [78], publication bias may not be serious. Egger’s regression test [79] also confirmed the conclusion (*p*s > 0.05).

None of these study moderators were found to be significant in Step 2 through Step 3. Consequently, Step 4 was skipped, and the unadjusted pooled correlation matrix generated in Step 1 was used in Step 5. Three models were tested and compared on their performance (*R*^2^). All variables directly predicted cancer-screening behavior in Model 1, while all variables predicted intentions and behavior simultaneously in Model 2. Model 2 was further simplified into Model 3, in which intentions fully mediated the effects of the independent variables on behavior. Through comparing changes in *R*^2^, Model 3 was found to be superior to both Model 2 and Model 1. In Model 3, the *R*^2^ values of behavior and intentions were 3% (4% in both Models 1 and 2) and 17% (12% in Model 2), respectively, which indicated a trivial amount of the explained variance of behavior but an acceptable amount of the explained variance of intentions. Furthermore, all of the fit indices of Model 3 met the cut-off criteria [80] ($\chi^{2}($7) = 41.723, *p* < 0.001, RMSEA (Root Mean Square Error of Approximation) = 0.011, RMSEA_lower 95% CI_ = 0.008, RMSEA_upper 95% CI_ = 0.014, SRMR (Standardized Root Mean Square Residual) = 0.040, CFI (Comparative Fit Index) = 0.947, AIC (Akaike Information Criterion) = 27.723, and BIC (Bayesian Information Criteria) = −32.807), indicating that the model fits the data well. It was found that the variable of intentions was only positively predicted by cues to action (β β = 0.236, *p* < 0.001), health literacy (β = 0.138, *p* < 0.001), and perceived susceptibility (β = 0.11, *p* < 0.05) and that behavior was negatively predicted by intentions (β = −0.130, *p* < 0.001) (see Table 2). All the other predictions were not significant. The total indirect effect was −0.149, which means that all of the predictors had trivial negative effects on behavior through intentions.

## 5. Discussion

### 5.1. Discussion of Estimation Results

We found that the individual importance of predictors of the health belief model (HBM) is unequal through MASEM. Cues to action, health literacy, and perceived susceptibility are the significant determinants of cancer-screening intention. The long-believed core components of the HBM, i.e., perceived barriers, perceived benefits, and perceived severity, played a marginal role in predicting intention. In addition, the added variable, i.e., perceived behavioral control, was also not a significant predictor of intention. This finding is generally consistent with the synthesis of Janz and Becker [57], who found that perceived benefits and perceived severity were strong only for sick-role behaviors (SRB) and that perceived susceptibility was a stronger contributor for explaining preventive-health behaviors (PHB) than SRB.

The finding of the three key predictors of screening intention demonstrates that internal cues to action (e.g., feeling hurt or other symptoms of getting cancer) and perceived susceptibility are directly related to immediate personal vulnerability or the probability of getting cancer. Moreover, internal cues and perceived vulnerability may be triggered or primed by external cues to action (e.g., the doctor’s warnings or a close relative’s death due to cancer). Furthermore, there is no salient or discernible imminent threat or risk without sufficient awareness and knowledge, i.e., health literacy [32].

Conversely, the insignificant effects of perceived barriers, perceived benefits, and perceived severity on screening intention indicate that general outcome expectancies or beliefs about screening benefits and barriers are too general and distant to concern and urge people [146], who do not act until they realize they are in real danger. The factor of perceived behavioral control or self-efficacy is not important to predict intention. Such a finding shows that the intended uptake of screening has nothing to do with ability, which is emphasized in the studies based on the theory of planned behavior (TPB). This finding implies that the application area of intentions may moderate the effects of the TPB predictors, and future research should examine such a hypothesis.

### 5.2. Gap between Health Intentions and Health Behaviors

This study found that the gap between intention and behavior is real, and this result is also consistent with part of the prior findings [147]. For instance, some researchers [54,56,83] found that the health belief model (HBM) was weakly predictive of behavior in comparison with social cognitive theories, such as the theory of planned behavior (TPB) and the theory of reasoned action (TRA) [55]. The present study has further discovered that the determinants of both intentions and behavior embodied in the TRA and the TPB also only partially predict intentions [148] and that the performance of a behavior is not as simple as a natural outcome of intentions [146,149]. The HBM (as well as other value-expectancy-based theories) is better represented as an effect of health beliefs on health intentions rather than behavior and hence is hardly a behavior prediction theory.

There are two possible paths to improve the prediction of behavior. The first is to strictly stick to the original or reconsider both the conceptualization and operationalization of factors encompassed in these theories. For instance, attitudes, subjective norms, and perceived behavioral control were often incorrectly directly measured and tested in many applied studies. In addition, as Fishbein and Cappella [146] pointed out, specific instead of general beliefs associated with screening characteristics ultimately underlie and determine intentions and behaviors. Some crucial determinants of health behavior have been notably unheeded in the literature, and hence they may be included to translate intentions into behavior. For instance, Gollwitzer [150,151] proposed the implementation of intentions to improve the prediction of actual behavior while arguing that individuals pass control to the environment, which acts as a cue to action. When cues are present, the performance of the intended behavior ensues [150]. Fishbein [146,149] incorporated actual ability and environmental factors to predict behaviors in addition to intentions. Wakefield, Loken and Hornik [9] underscored policy support. Furthermore, screening intentions may be temporal and situational [148]. Consequently, some authors [152,153] combined the HBM and the transtheoretical model (TTM) (or stages of change) in their screening research, whereas others [154] further incorporated the dual-process model in the combination of the HBM and TTM. Moreover, suggesting a distinction between pre-intentional motivation processes that lead to a behavioral intention and post-intentional volition processes that lead to actual behavior, Schwarzer and his associates [155,156] proposed the health action process approach (HAPA) by incorporating three post-intentional factors (planning, maintenance self-efficacy, and action control) to the expanded HBM. These efforts are commendable, but the effect should also be amenable to a model-based meta-analysis.

## 6. Implications

### 6.1. Theoretical Implications

**Figure 5:**
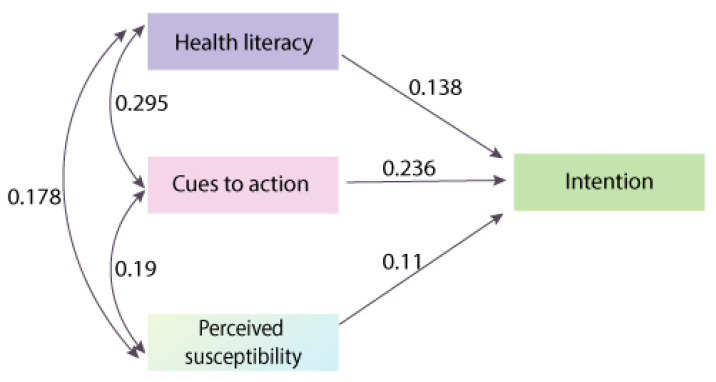
The health intention model.

Given the estimation results of path analysis, a model predicting screening intentions, termed the health intention model, is proposed (see Figure 5). Our proposed model is much more parsimonious (only retains the two original variables, i.e., cues to action and perceived susceptibility) than the HBM and slightly expands the HBM through the addition of health literacy. The model was tested with a perfect model fit (the RMSEA and RSMR were ~0, and the CFI was ~1), and all predictors had significant positive effects on intentions (*p*s < 0.001, *R*^2^ = 13%). Although the proposed parsimonious model is open to further tests, it has a very similar explanatory power to the complex HBM, albeit with significant predictions on intentions using the collective data source. Consequently, the present study has significantly advanced the development of theories on cancer-screening intentions.

### 6.2. Practical and Methodological Implications

The findings also have profound practical implications. Health communication campaigns should focus on enlightening and engaging the public through all necessary means to raise awareness and transfer knowledge in relation to screening procedures as well as cancers per se. Nevertheless, the downside of the finding is that the knowledge gap [157] or inequality in health literacy is pervasive among ethnicities and various social strata [158,159] and is hence a thorny worldwide problem to tackle. Therefore, to improve universal health literacy, health communication has a long way to go.

To our knowledge, the present paper is one of the very few model-based meta-analytical studies in health communication, although Eisend [160] briefly summarized the gist of MASEM, and James [161] applied MASEM in an interdisciplinary journal. This study has also discovered the limitations of the HBM and other similar value-expectancy-based theories in predicting cancer-screening intentions and behavior. As a result of this finding, a parsimonious model is proposed, and other remedies to the existing models are also discussed. Moreover, effective health communication strategies and interventions are suggested. Therefore, the present study has made substantial theoretical, methodological, and practical contributions to health communication.

## 7. Limitations and Future Research Directions

This study has limitations. Since the model-based meta-analysis requires complete correlations, some meaningful predictors, such as trust in health providers, social capital, and demographics, were removed in the present study. This constraint has prevented us from exploring more possibilities of theory testing as well as advancement. An additional limitation results from the developmental nature of the model-based meta-analysis methodology, whose algorithms and software packages are works in progress. As a result, some forms of theoretical explorations may not be available.

Moreover, we only considered the studies that examined the positive cancer-screening intentions and behaviors but neglected those that studied overscreening behaviors. Nevertheless, overscreening has psychological and social implications [162]. A future meta-analysis should include studies in the two separate lines of research and ideally examine the differences in predictors and effect sizes.

## Figures and Tables

**Table 1:** Pooled zero-order correlation matrix.

	PBAR	PBEN	PBC	INT	PSEV	PSUS	CTA	CS	LIT
PBAR	1	51 (53,317)	73 (64,065)	86 (62,920)	31 (19,138)	45 (41,254)	44 (33,560)	129 (67,178)	65 (38,460)
PBEN	−0.123 ***	1	19 (31,881)	21 (35,175)	12 (8412)	20 (25,018)	12 (15,568)	20 (18,416)	19 (9630)
PBC	−0.09 ***	0.279 ***	1	32 (39,986)	8 (6359)	15 (20,030)	14 (17,779)	42 (30,184)	20 (16,721)
INT	−0.116 ***	0.267 ***	0.212 ***	1	10 (5389)	25 (20,021)	33 (20,319)	26 (26,423)	34 (16,113)
PSEV	−0.016	0.081	0.009	0.029	1	10 (7505)	9 (3922)	16 (6106)	10 (7344)
PSUS	−0.043 *	0.183 ***	0.038	0.181 ***	0.197 ***	1	9 (9555)	25 (18,411)	18 (16,883)
CTA	−0.086 ***	0.103 *	0.087 ^#^	0.166 ***	0.01	0.16 *	1	36 (24,323)	14 (9225)
CS	−0.147 ***	0.196 ***	0.164 ***	0.259 ***	0.031	0.071 **	0.167 ***	1	50 (28,376)
LIT	−0.05 *	0.175 ***	0.172 ***	0.158 ***	0.008	0.045	0.072	0.145 ***	1

Note: *** *p* < 0.001, ** *p* < 0.01, * *p* < 0.05, ^#^ *p* < 0.10. CS = screening behavior; INT = intention; CTA = cues to action; LIT = health literacy; PBAR = perceived barriers; PBC = perceived behavioral control or self-efficacy; PBEN = perceived benefits; PSEV = perceived severity; and PSUS = perceived susceptibility. The numbers shown in the upper triangular matrix represent the k (number of effect sizes) and the total sample size (within brackets).

**Table 2:** Results of the WLS estimation using Metasem.

	Estimate	Std. Error	z Value	Pr (>|z|)
CS on INT	−0.13	0.041	−3.203	0.001
INT on CTA	0.236	0.027	8.673	0
INT on LIT	0.138	0.035	3.981	0
INT on PBAR	−0.012	0.047	−0.253	0.801
INT on PBC	−0.03	0.074	−0.409	0.683
INT on PBEN	0.039	0.044	0.903	0.367
INT on PSEV	0.093	0.071	1.319	0.187
INT on PSUS	0.11	0.056	1.972	0.049

Note: *n* = 42,071, $\chi^{2}$(7) = 41.723, *p* < 0.001, RMSEA = 0.011, RMSEA_lower 95% CI_ = 0.008, RMSEA_upper 95% CI_ = 0.014, SRMR = 0.040, TLI = 0.726, CFI = 0.947, AIC = 27.723, and BIC = −32.807. CS = screening behavior; INT = intention; CTA = cues to action; LIT = health literacy; PBAR = perceived barriers; PBC = perceived behavioral control or self-efficacy; PBEN = perceived benefits; PSEV = perceived severity; and PSUS = perceived susceptibility.
